# Natural Killer Cell Engagers (NKCEs): a new frontier in cancer immunotherapy

**DOI:** 10.3389/fimmu.2023.1207276

**Published:** 2023-08-09

**Authors:** Minchuan Zhang, Kong-Peng Lam, Shengli Xu

**Affiliations:** ^1^ Singapore Immunology Network, Agency for Science, Technology, and Research, Singapore, Singapore; ^2^ Department of Microbiology and Immunology, Yong Loo Lin School of Medicine, National University of Singapore, Singapore, Singapore; ^3^ School of Biological Sciences, College of Science, Nanyang Technological University, Singapore, Singapore; ^4^ Department of Physiology, Yong Loo Lin School of Medicine, National University of Singapore, Singapore, Singapore

**Keywords:** cancer, immunotherapy, natural killer (NK) cell, NK cell engager (NKCE), functionality, manufacturability

## Abstract

Natural Killer (NK) cells are a type of innate lymphoid cells that play a crucial role in immunity by killing virally infected or tumor cells and secreting cytokines and chemokines. NK cell-mediated immunotherapy has emerged as a promising approach for cancer treatment due to its safety and effectiveness. NK cell engagers (NKCEs), such as BiKE (bispecific killer cell engager) or TriKE (trispecific killer cell engager), are a novel class of antibody-based therapeutics that exhibit several advantages over other cancer immunotherapies harnessing NK cells. By bridging NK and tumor cells, NKCEs activate NK cells and lead to tumor cell lysis. A growing number of NKCEs are currently undergoing development, with some already in clinical trials. However, there is a need for more comprehensive studies to determine how the molecular design of NKCEs affects their functionality and manufacturability, which are crucial for their development as off-the-shelf drugs for cancer treatment. In this review, we summarize current knowledge on NKCE development and discuss critical factors required for the production of effective NKCEs.

## 1 Introduction

Immunotherapy has profoundly impacted cancer treatment, spurring a multi-billion-dollar industry devoted to discovering and developing novel immunotherapeutic drugs. Unlike conventional chemotherapy or radiotherapy, which directly targets tumor cells, immunotherapy harnesses immune cells to combat cancer. Therapeutic antibodies against immune checkpoint receptors, such as PD-1 and CTLA-4, along with adoptive transfer of genetically engineered chimeric antigen-receptor (CAR)-T cells, have demonstrated tremendous success in treating some cancers. Specifically, checkpoint inhibitors have been notably effective against skin and lung cancers, while CAR-T therapy has been remarkably successful in the treatment of some haematological malignancies, with both unleashing the full antitumor activities of T cells (1).

While T-cell immunotherapies exhibit remarkable advantages, they also present some constraints and limitations. For example, although anti-PD-1 and anti-CTLA-4 monoclonal antibodies show spectacular clinical outcomes (>70% efficacy) against cancers such as melanoma, they can only achieve less than 20-30% efficacy in most other cancer types, exhibiting considerable variability among patient responses and often leading to relapse in initial responders (2, 3). Likewise, the USA FDA-approved CAR-T cell therapy, such as Yescarta and Kymriah, was initially perceived as a medical miracle as a single dose could cure certain blood cancers. Still, ongoing research uncovers several challenges, including disease relapse, limited success in solid tumors, and severe side effects such as cytokine release syndrome (CRS) and neurotoxicity, which hinder their broad adoption (4–7). Additionally, CAR-T therapy is limited by the quantity and quality of autologous T cells obtained from cancer patients, who are often immune compromised as a result of the disease or treatments. The prolonged and complex process of scaling up the production of autologous CAR-T cells for patients with limited life span and its daunting price tag also pose significant hurdles (8, 9).

These challenges underscore the urgent need to develop new immunotherapeutic modalities and the exploration of other types of immune cells, such as Natural Killer (NK) cells. NK cells could potentially circumvent some of the inherent limitations of T-cell immunotherapy. As one of the initial defences in antitumor immunity, NK cells naturally possess the ability to kill tumor cells without prior sensitization and modulate antitumor functions via cytokine secretion (10). Therapeutic antibodies inducing NK cell antibody-dependent cellular cytotoxicity (ADCC) and genetically engineered CAR-NK cells have been developed for cancer treatment. However, these two modalities bear some inherent limitations. For instance, polymorphism at positions 48 and 158 of CD16a has been reported to impact human IgG1 binding and subsequently influence NK cell ADCC (11–14). Moreover, it is well-known that CAR-NK cells present significant challenges in genetic manipulation, and their antitumor efficacy is limited by the short life of NK cells (15, 16). Another class of immunotherapeutic modalities harnessing NK cells, known as NK cell engagers (NKCEs), has recently emerged and exhibited several advantages over NK cell ADCC and CAR-NK cell strategies. In this review, we will discuss various aspects with regard to NKCE development, including the selection of target molecules for NK activation by NKCEs, the design of NKCE formats, and factors impacting the functionality and manufacturability of NKCEs. Moreover, we aim to illuminate the challenges and opportunities in developing NKCEs as potent immunotherapeutics for cancer treatment.

## 2 Biology of NK cells

NK cells are a type of innate immune cells belonging to the group 1 innate lymphoid cell (ILC) family. They play a crucial role in the body’s first line of defence by identifying and eliminating stressed cells, including those infected by viruses and tumor cells (17, 18). Unlike T cells, NK cells do not express CD3; instead, they express CD56 on their cell surface. They constitute 5% to 15% of circulating lymphocytes in the blood (19). NK cells can be categorized into two major subsets based on their CD56 and CD16a expression: (1) immature CD56^bright^CD16a^dim^ NK cells, known for their high production of cytokines, such as IFNγ, and (2) mature CD56^dim^CD16^bright^ NK cells, making up 90% of peripheral blood NK cells, primarily exerting cytotoxic function (19).

NK cells recognize membrane-bound ligands on target cells through an array of germline-encoded activating and inhibitory receptors on their cell surface (17). The activation status of NK cells is determined by the balance of activating and inhibitory signals they receive upon contact with other cells. Major histocompatibility complex class I molecules (MHC I)/Human leukocyte antigens (HLA), which are abundantly expressed on healthy cells, inhibit NK cell activation through the killer immunoglobulin-like (KIR) family of inhibitory receptors present on the surface of NK cells. Conversely, NK cells become activated when there is a downregulation of HLA molecules on the target cells or an upregulation of the ligands for NK activating receptors, such as NKp46 and NKG2D. NK cells also get activated when their CD16a receptors are engaged by antigen-bound IgGs. When activating signals prevail over inhibitory signals, NK cells become activated and release cytotoxic granules loaded with perforin and granzymes, causing target cells to lyse. In addition, NK cells can induce apoptosis of target cells through Fas-L or TRAIL (20), especially during the later phases of NK cell serial killing (21).

Compared to T cells, NK cells have several unique advantages in the context of immune cell therapy. As they are not HLA-restricted, NK cells can be sourced allogenically, thereby making them a feasible “off-the-shelf” therapeutic option. KIR-ligand-mismatch between donors and recipients can even potentially enhance the effectiveness of adoptive NK cell therapy by circumventing inhibitory signals on host cells (22, 23). Additionally, unlike T cells, NK cells do not secrete high levels of cytokines that could trigger CRS. They also do not cause graft-versus-host (GVH) reactions, making them a safer treatment option. As of 2021, more than 400 reported clinical trials for cancer treatment based on NK cells have been reported (24) including monotherapies involving NK cells from various sources, such as peripheral blood,umbilical cord blood, hematopoietic stem cell-derived NK, and CAR-NK cells (24). Additionally, combination therapies have been explored, which pair NK cells with other therapeutic agents, such as immune checkpoint inhibitors, antibodies or NK cell engagers (24).

## 3 Cancer immunotherapies directing NK cells

**Figure 1:**
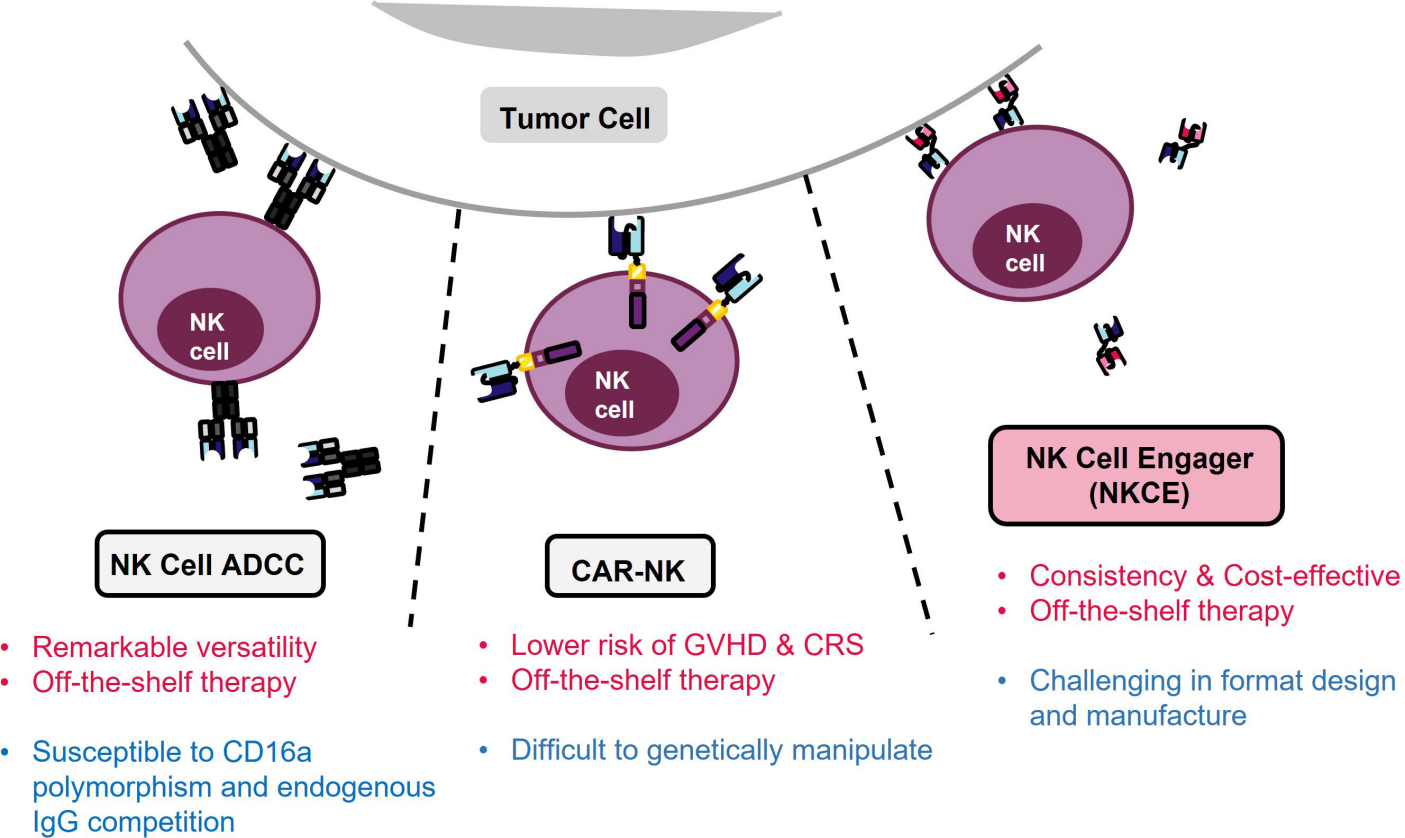
Three strategies for NK immunotherapy. Pros of each strategy are denoted in red, and limitation is marked in blue. NK ADCC targets tumor cells using monoclonal antibodies (mAbs) and activates NK cells through the CD16a Fc receptor. However, the efficacy of NK ADCC can be reduced by polymorphism of CD16a, resulting in lower binding of mAbs. In contrast, CAR-NK therapy avoids the problems associated with CD16a polymorphism but it suffers from difficulties in genetic manipulation of NK cells. On the other hand, NKCE offers a balanced profile and a cost-effective solution for NK cell-based cancer immunotherapy.

There are three primary approaches directing NK cells for cancer treatment: 1) employing NK cell antibody-dependent cellular cytotoxicity (NK cell ADCC), which is initiated through the Fc receptor CD16a on NK cells and directed by a monoclonal antibody (mAb); 2) genetically engineering CAR-NK cells that specifically target tumor antigens using the CAR and become activated; 3) developing recombinant proteins, known as NK cell engagers (NKCE), that bring tumor and NK cells into proximity and activate the NK cells (**Figure 1**).

NK cell ADCC leverages the antigen-specificity of mAbs to redirect NK cells towards tumor cells. This approach relies on the binding of the Fc region of the mAbs to the Fcγ receptor IIIa (or CD16a) of NK cells, thereby triggering CD16a activation signalling and inducing the lysis of the mAb-coated target cells by NK cells (25). Currently, there are many mAbs available on the market that target different cancers, such as Trastuzumab, Pertuzumab, and Margetuximab for HER2^+^ cancers (25), Rituximab for non-Hodgkin’s lymphoma (26), and Cetuximab for EGFR^+^ colorectal cancer (27). The versatility of mAbs positions the NK cell ADCC method as an effective approach for various cancer treatments. However, not all patients respond well to this approach. The presence of the 158F variant of CD16a can decrease its binding affinity for IgG mAbs, which is the primary isotype of therapeutic mAbs (28, 29). Remarkably, a majority of the population carries at least one *CD16a* allele of this variant (15), which could potentially undermine the effectiveness of NK cell ADCC treatment (28, 30). Nevertheless, the issue of CD16a polymorphism could potentially be mitigated through afucoslylation or engineering of the Fc region of mAbs to enhance their binding affinity to CD16a (31).

CAR-NK cell therapy can circumvent the issue of CD16a polymorphism present in NK cell ADCC as the genetically engineered CAR-NK cells recognize and target cancer cells using their antigen-specific CARs, which signal and activate NK cells through their signalling module-bearing cytoplasmic tails (32). Compared to CAR-T cell therapies, CAR-NK cells can be produced more efficiently from an allogenic source (32). Additionally, CAR-NK therapy is safer due to the lower risk of GVHD and CRS (33). A variety of CAR-NK cells are currently undergoing assessment for their efficacy against haematological and solid tumors in both preclinical and clinical studies (24, 32, 34).

Despite its promising potential, CAR-NK cell therapy is still in the early stage of development, with many challenges yet to be addressed. A key issue is the short lifespan of CAR-NK cells, which do not persist in the body for long (35). This necessitates frequent infusion, thereby increasing the treatment costs (32). Therefore, it is crucial to find a solution to extend the *in vivo* survival of CAR-NK cells. Furthermore, CAR-NK cells present more significant challenges in genetic manipulation compared to CAR-T cells. Their lower transduction efficiency of NK cells necessitates a larger initial NK cell number, followed by sorting and expansion (34), increasing the overall costs and extending the production time.

NKCEs, such as BiKE (bispecific killer cell engager) or TriKE (trispecific killer cell engager), provide several advantages over the earlier two strategies. Compared to bispecific BiKEs, TriKEs possess an additional antigen specificity, enabling more precise targeting and enhanced functionalities. In contrast to tumor-targeting mAbs, CD16a-targeted NKCEs are not necessarily affected by the CD16a polymorphism, as they may recognize regions not influenced by the polymorphism (36). Additionally, NKCEs engaging activating receptors on NK cells other than CD16a are not affected by CD16a polymorphism or its expression level. Unlike in the case of NK cell ADCC, these NKCEs do not compete with endogenous IgG1 for CD16a binding. Besides activating NK cells, NKCEs could also activate other immune cells and redirect them towards tumor cells if they target receptors commonly expressed in NK and other immune cells. For example, NK cell activating receptor NKG2D is also expressed in NKT, some CD8^+^ T and a subset of γδ T cells (37), thus NKG2D-targeting NKCEs can potentially activate these immune cells in addition to NK cells. Moreover, NKCEs are easier to manufacture and much less expensive than CAR-NK cell therapy. Appropriately designed NKCEs also have an extended retention time in the body (38), making NKCEs a more cost-effective strategy compared to CAR-NK cell therapy (34).

NKCEs can also be combined with adoptive NK cell transfer by forming complexes with allogenic NK cells (15, 39). A study combining AFM13 and cord-blood NK cells has shown that this combination stimulates the NK cells against CD30^+^ tumor cells (40). This approach circumvents the need for genetic engineering of CAR-NK cells, as retaining NKCEs on the NK surface eliminates the requirement for recurrent genetic modifications of NK cells (41, 42). Several clinical studies are currently in progress to assess the effects of co-administering adoptive NK cell CYNK-101, an NK cell line optimized for IgG binding, with anti-CD38 mAb Daratumumab (43) or anti-HER2 Trastuzumab and Pembrolizumab (NCT05207722) for cancer treatment.

Although NKCEs offer several advantages as an immunotherapeutic modality, substantial efforts towards optimization are required to develop potent and effective NKCEs. Therefore, several critical factors must be carefully considered during NKCE development.

## 4 Developing potent NKCEs by targeting various NK cell receptors

### 4.1 Engaging conventional activating receptors on NK cells

#### 4.1.1 CD16a

CD16a, also known as Fcγ receptor IIIa (FcγRIIIa), is an activating receptor abundantly expressed in CD56^dim^ NK cells. It is anchored to the plasma membrane of NK cells and is associated with CD3ζ and/or FcϵRIγ chain (44) (**Table 1**). Upon engagement by IgG antibodies, CD16a molecules cluster together, leading to the phosphorylation of the tyrosine residues of the ITAM motifs of CD3ζ and/or FcϵRIγ chains by Src-family kinases. Subsequently, signalling cascades involving phosphatidyl-inositol-3-OH kinase (PI3K), phospholipase C γ (PLCγ) (45), and Vav (46) are triggered in NK cells. Unlike other NK cell activating receptors that require co-engagement of additional receptors to achieve optimal activation, engagement of CD16a alone can fully activate NK cells (47). The full activation of NK cells by CD16a engagement also differs from the optimal activation of T cells, which necessitates the ligation of both T cell receptors and co-stimulatory receptors. Certain bispecific T cell engagers (BiTEs), which require engagement of the costimulatory receptor CD28 to fully activate T cells and eliminate tumor cells (48). Therefore, NKCEs that engage CD16 are capable of inducing complete activation of NK cells without the need for triggering other NK cell activating receptors. 

**Table 1:** The structures and properties of NK cell activating and inhibitory receptors targeted by NKCEs.

Name	Structure	Advantages	Limitations
CD16a	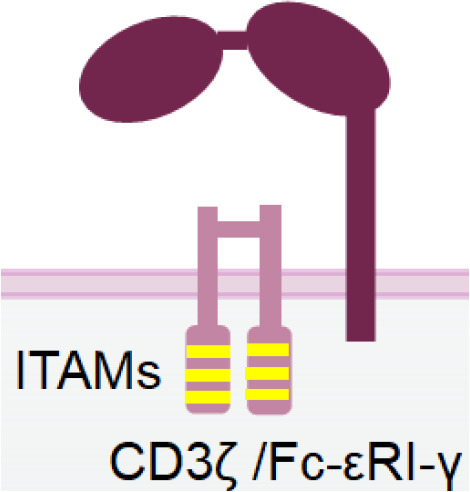	Inducing strong activation	Decreased surface expression upon NK cell activation
NKG2D	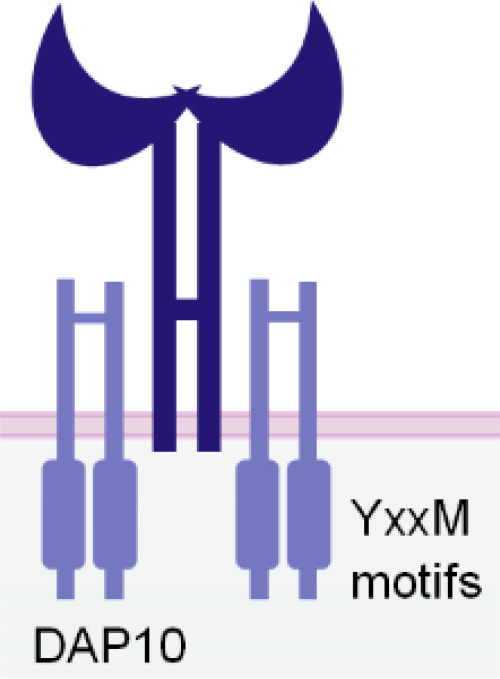	Expression in both NK and CD8^+^ T cells Triggering rapid NK cell activation	Inducing weaker activation than CD16a
Nkp30	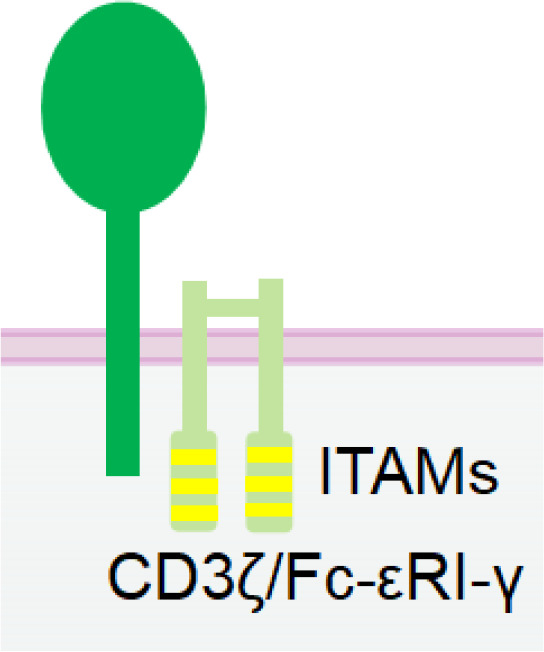	Constitutive expression in NK cells	Low expression in NK cells Inducing weaker activation than CD16a
Nkp46	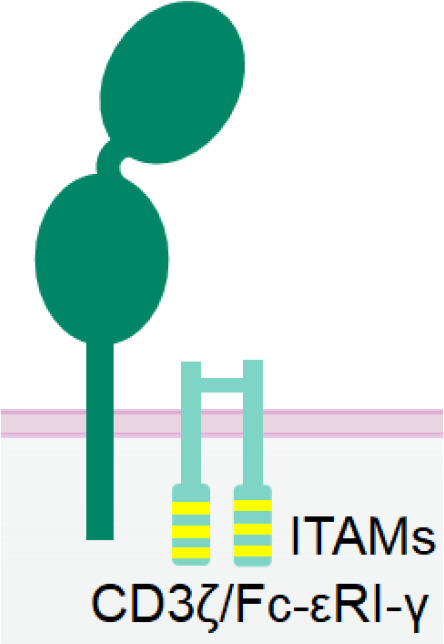	Specific and constitutive expression in NK cells	Inducing weaker activation than CD16a
Nkp80	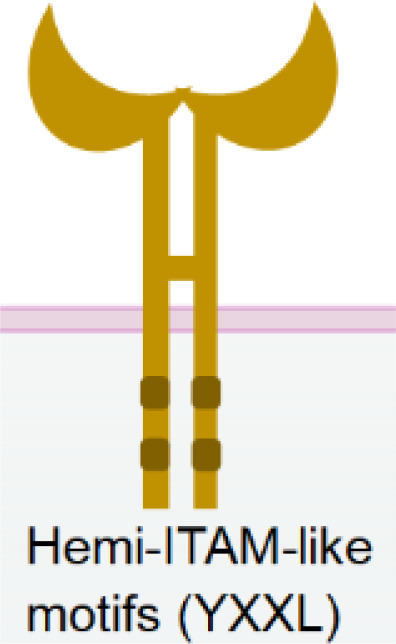	Specific expression in NK cells	Lower expression in NK cells
NKG2C/CD94	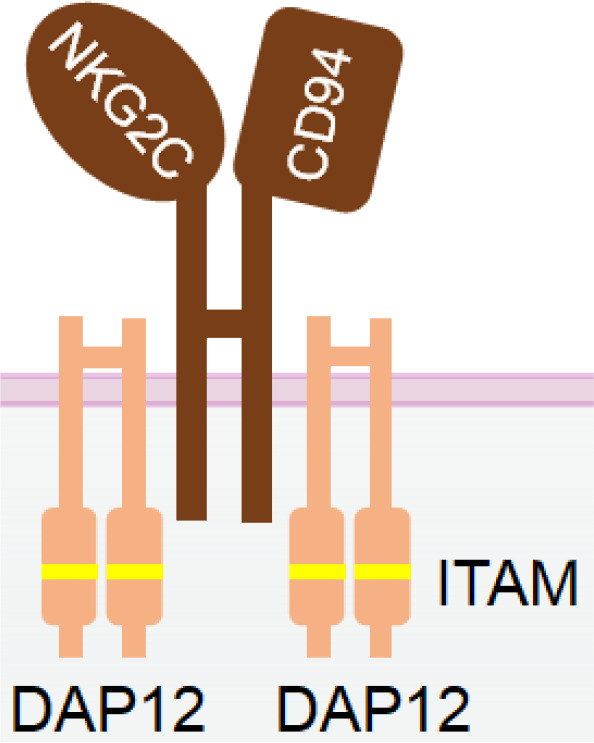	Signifying a "memory" NK cell phenotype Inducing strong activation	Lower and variable expression in NK cells
KIR2DS/KIR3DS	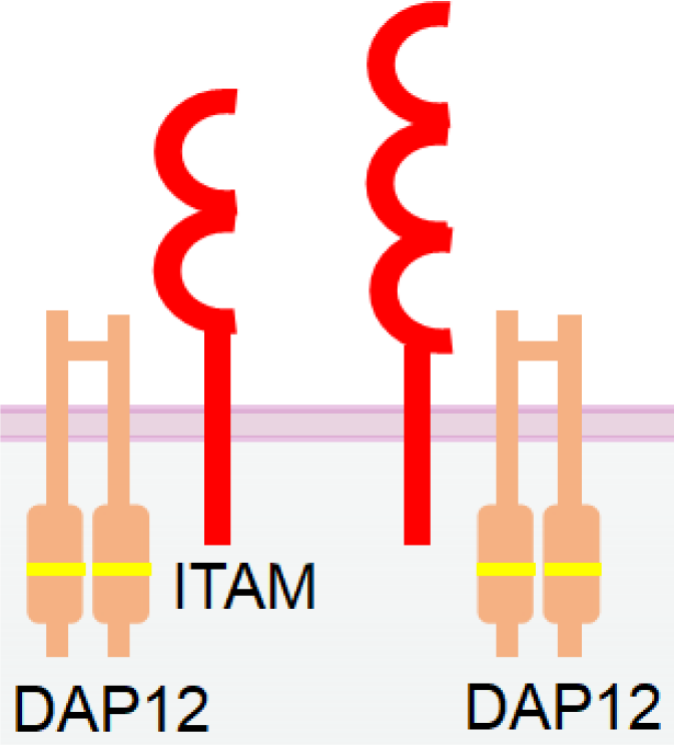	Constitutive expression in NK cells	High homology to inhibitory KIRs
CD160	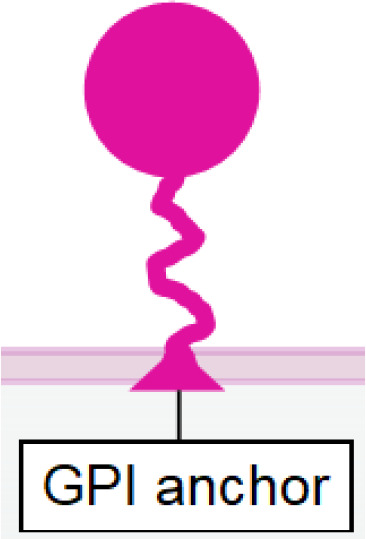	Expression in both NK and CD8^+^ T cells	Downregulation upon NK cell activation
DNAM-1	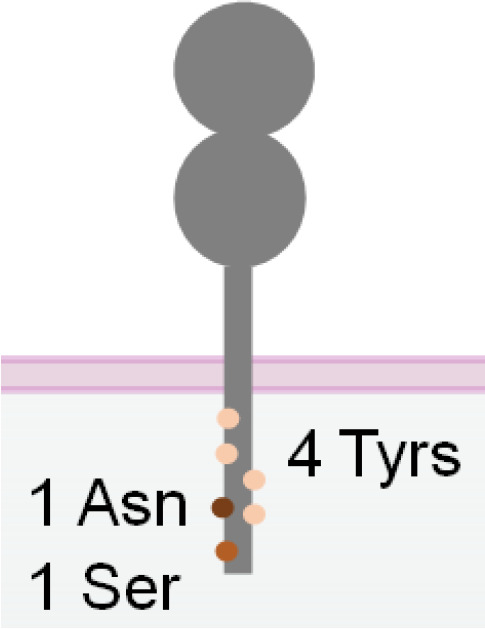	Promoting NK cell adhesion to target cells	Inability of activating NK cells directly
2B4	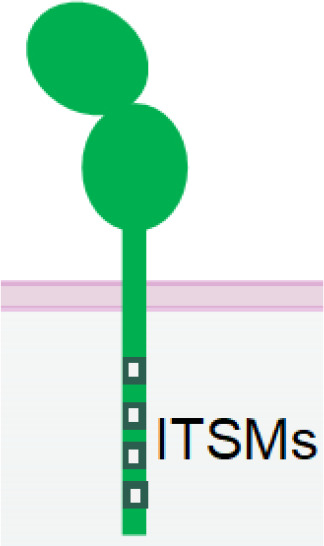	Inducing strong activation by synergizing with other activating receptors	Inability of activating NK cells directly
IL-2/IL-15	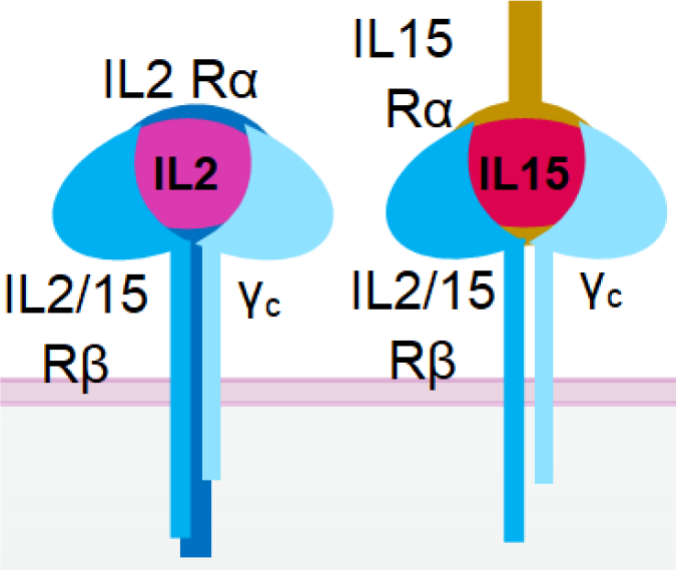	Enhancing NK cell proliferation and cytotoxicity	Inability of activating NK cells directly Unspecific activation of Treg by IL-2
PD-1	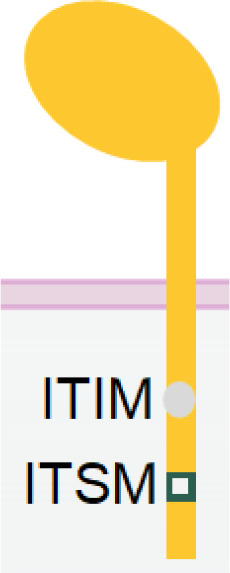	Antagonizing NK cell and CD8^+^ T cell exhaustion by receptor blockade	Inability of activating NK cells directly Requirement of high dosage for effective blockade
NKG2A	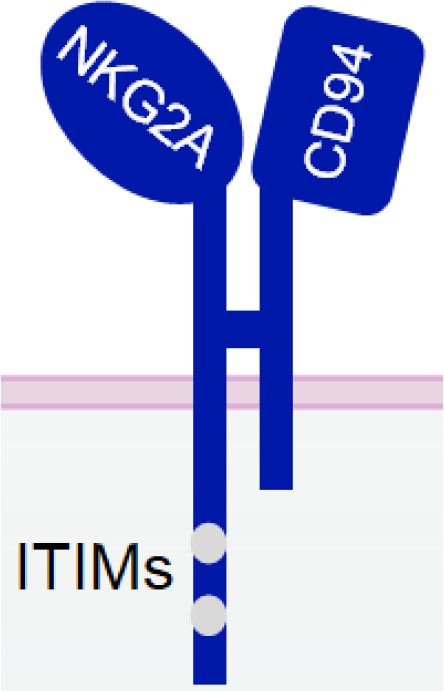	Antagonizing NK cell and CD8^+^ T cells exhaustion by receptor blockade	Inability of activating NK cells directly
TIGIT	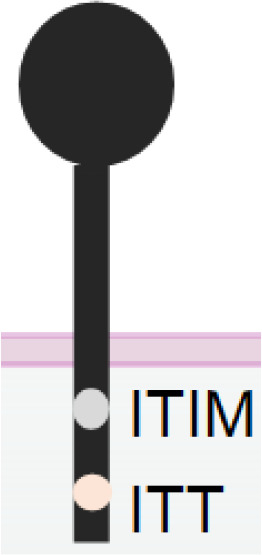	Antagonizing NK cell exhaustion by receptor blockade Combinatory therapy with the PD-1 blockade	Inability of activating NK cells directly
TIM-3	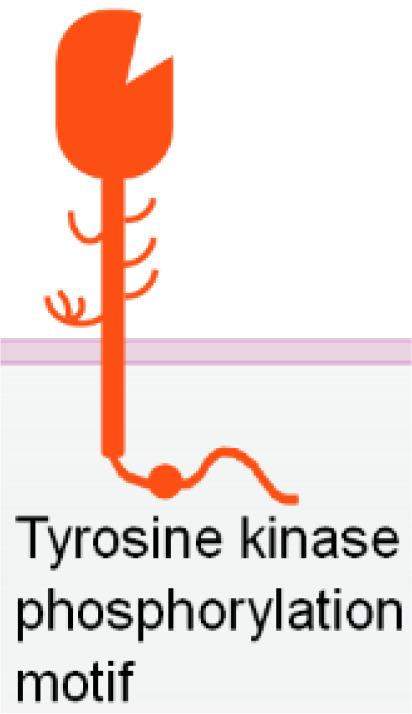	Antagonizing NK cell exhaustion by receptor blockade	Inability of activating NK cells directly
KIR2DL/KIR3DL	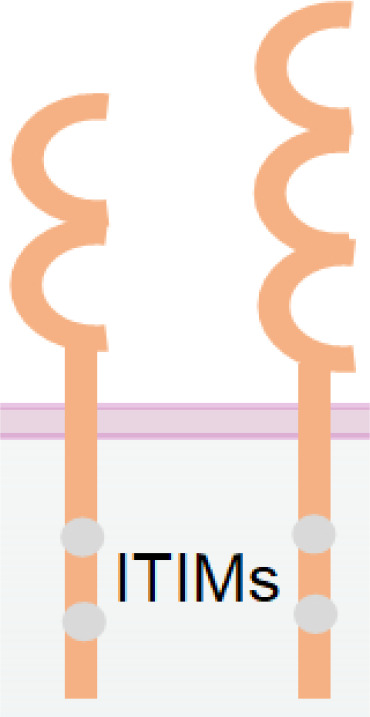	Enhancing NK cell activation by receptor blockade	Inability of activating NK cells directly
CD96 (TACTILE)	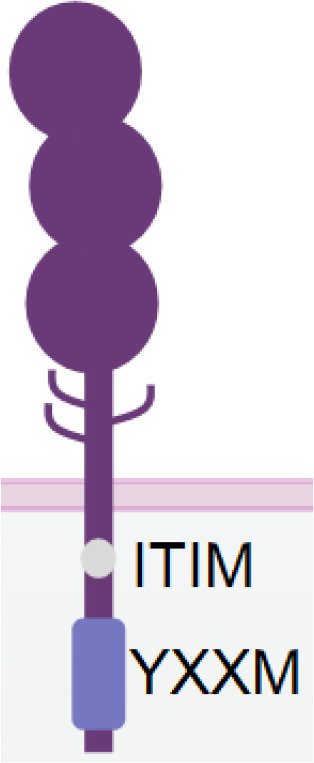	Antagonizing NK cell exhaustion by receptor blockade Combinatory therapy with the PD-1 blockade	Inability of activating NK cells directly

Different receptors have distinct advantages and limitations when used as NKCE targets. CD16a has strong activation strength but suffers from downregulation in activated NK cells. Cytokines IL-15 and IL-2 can enhance NK cell activity greatly but require specific delivery to NK cells, which can be facilitated by NKCEs. Nkp30 and Nkp46 are constitutively expressed on activated NK cells which makes the NKCE has longer effects, but they may need a co-activation receptor for stronger NK activation. NKG2D NKCE has the potential to recruit CD8+ T cells and thus further boost tumor cell killing. Moreover, blockade of immune checkpoint receptor like PD-1, TIGIT or CD96 etc. on NK cells can recover NK from exhaustion, but the immune checkpoint blockade strategies are not able to activate NK cells directly.

**Figure 2:**
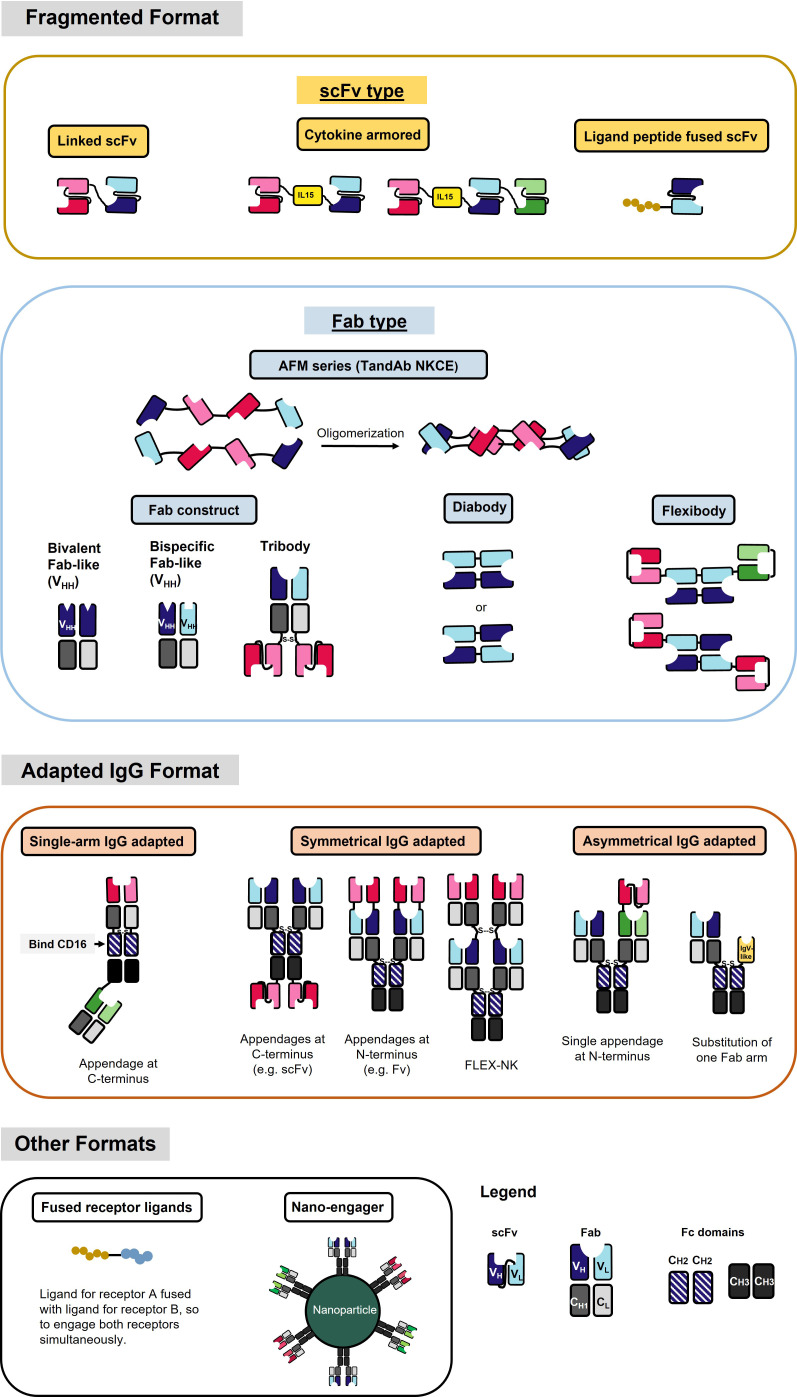
NKCE formats that are currently under development. Different color (red, blue, and green) indicates different antigen specificity. The dark and light colors label the heavy chain (VH) and light chain (VL), respectively. The fragmented format contains a variety of structures by different linkages of the VH and VL, including scFv and Fab. The adapted IgG format contains structures that generally have the constant region of the IgG antibody. Single-arm IgG adapted format uses Fc region CH2 domain to bind to CD16a. Please refer to **Table 2** for specific NKCEs.

Currently, CD16a is the most popular target for NKCE development, with many products currently in preclinical development or clinical trials (**Table 2**). For example, AFM13, a tetravalent bispecific anti-CD30xCD16a NKCE, is formed by homodimerization of a tandem diabody (TandAb), which is constructed through a special arrangement of Fv heavy and light chain domains of anti-CD30 and -CD16a antibodies with a 9-amino acid-long peptide linker (**Table 2** and **Figure 2**). This 104 kDa TandAb has demonstrated CD30-dependent activation of NK cells, with a half-life of 19 hours (68). Clinical trials have shown AMF13 to be effective in treating patients with relapsed or brentuximab vedotin-refractory Hodgkin’s lymphoma, and it was well-tolerated during continuous treatment (68, 91, 92). With the proven effectiveness of AFM13, NKCEs with similar structures (AFM22/24/26) (**Table 2**) have also been developed to target EGFR vIII, EGFR, and BCMA, respectively (93, 94). AFM22 and AFM24 are designed for the treatment of solid tumors. AFM24 is currently undergoing a Phase 1/2a clinical trial for advanced solid cancers (NCT04259450) and is also being tested in combination with Atezolizumab for EGFR-expressing advanced solid malignancies (NCT05109442). On the other hand, AFM22, which specifically targets EGFR vIII is still in the preclinical development stage. In addition, preclinical evaluation of BCMA-targeted AFM26 has shown that it binds to NK cells with high avidity and is largely unaffected by low BCMA expression levels on multiple myeloma (MM) cells (94).

**Table 2:** NKCEs that are currently undergoing development.

Category	Name	Format	Cancer type	Reference
Linked scFv	CD16axEpCAM	Linked scFv, HMA linker	Carcinoma	(49)
	CD16axCD33	Linked scFv	Myelodysplastic syndromes; Refractory Acute Myeloid Leukemia.	(50, 51)
	CD16axCD19	Linked scFv	non-Hodgkins lymphoma	(52)
	CD16axCD19xCD22	Linked scFv, HMA linker	non-Hodgkins lymphoma	(52)
	CD16axCD133	Linked scFv, HMA linker	Colorectal Cancer	(53)
	NKG2DxCS1	Linked scFv, HMA linker	Multiple myeloma	(54)
Linked VHH	CD16AxEGFR	Linked V_HH_	EGFR^+^ cancers, e.g Lung cancer	(55)
	CD16axIL-15xHER2 (CAM1615HER2)	Linked V_HH_ & scFv	Ovarian cancer	(56)
	CD16axHER2	Linked V_HH_ via HMA linker	Breast cancer	(36)
	CD16axIL-15xB7H3 (CAM1615B7H3)	Linked V_HH_ & scFv	Ovarian carcinoma	(57)
Linked affibody	CD16axBCMA	Linked non-immunoglobulin affibody affinity proteins	Multiple myeloma	(58)
Linked scFv with cytokine	1615133 TriKE (CD16axIL-15xCD133)	Linked scFv plus cytokine motif	Carcinoma	(59)
	CD16axIL-15xEpCAMxCD133	Linked scFv plus cytokine motif, Linkers: a 20aa segment of human muscle aldolase (HMA), EASGGPE, and mutated IgG hinge	Carcinoma	(60)
	CD16axIL-15xCLEC12A (CLEC12A TriKE)	V_HH_ & scFv plus cytokine motif	Acute myeloid leukemia	(61)
	NKG2CxIL-15xCD33	Linked scFv plus cytokine motif	Myeloid leukemia	(62)
	161519 (CD16axIL-15xCD19)	Linked scFv plus cytokine motif	Chronic lymphocytic leukemia; non-Hodgkin’s lymphoma	(63, 64)
	GTB-3550/161533 TriKE (CD16axIL-15xCD33)	Linked scFv plus cytokine motif	CD33^+^ malignancies, e.g Systemic mastocytosis, Acute myeloid leukemia	(38, 65)
Ligand peptide fused with scFv	AICLxHER2	Extracellular domain of ligand fused to HER2-scFv	Breast cancer	(66)
	B7-H6xHER2	Extracellular domain of ligand fused to HER2-scFv	Breast cancer	(66)
	ULBP2xHER2	Extracellular domain of ligand fused to HER2-scFv	Breast cancer	(66)
	PVRxHER2	Extracellular domain of ligand fused to HER2-scFv	Breast cancer	(66).
	2A9-MICA (MICAxBCMA)	Extracellular domain of ligand fused to BCMA-scFv	Multiple myeloma	(67)
Fab	AFM13 (CD16axCD30)	Tand Ab.	CD30^+^ malignancies, e.g Hodgkin lymphoma	(40, 68, 69)
	AFM22 (CD16ax EGFRvIII)	Tand Ab.	EGFRvIII malignancies: certain solid tumors e.g. glioblastoma, prostate cancer and head and neck cancer	Affimed
	AFM24 (CD16axEGFR)	Tand Ab. (AFM24_T) & Symmetrical adapted IgG1 antibody with C-terminal appendage (AFM24_I)	EGFR^+^ cancers, e.g Lung cancer	AACR 2020 Jun 22-24 Poster 5659 – AFM24, Affimed Company; (70); NCT04259450
	AFM26 (CD16axBCMA)/RO7297089	Tand Ab. & Symmetrical adapted IgG1 antibody with C-terminal appendage	Multiple myeloma	(71–73)
	(HER2)_2_xCD16a	Tribody	Breast cancer	(74)
	CD16axEGFR	Diabody	EGFR^+^ cancers, e.g Lung cancer	(75)
	CD16axCD200xBCMA	Flexibody	Multiple myeloma	(76, 77)
	NKG2DxHER2	Bispecific (bsFab) or bivalent Fab-like (bvFab) antibodies with V_HH_	Breast cancer	(78)
	NKG2DxFMDV	Bispecific (bsFab) or bivalent Fab-like (bvFab) antibodies with V_HH_	Foot-and-Mouth Disease Virus	(78)
Asymmetrical single-armed IgG-adapted	Nkp46xTumor Ag	Single-armed IgG-adapted	*Not specified*	(79)
	NKp46xCD19/20	Single-armed IgG-adapted	B-cell Precursor Acute Lymphoblastic Leukemia	(80)
	NKp30xCD19/20	Single-armed IgG-adapted	B-cell Precursor Acute Lymphoblastic Leukemia	(80)
Asymmetrical IgG adapted	CD16axEGFRxPD-1	Asymmetrical adapted IgG	EGFR^+^ cancers, e.g Lung cancer	(81)
	B7-H6xEGFR	Asymmetrical adapted IgG: 1 Fab is replaced by Ig-like V-type domain of the extracellular region of B7-H6	EGFR^+^ cancers, e.g Lung cancer	(82)
Symmetrical IgG adapted	AFM28 (CD16axCD123)	Symmetrical adapted IgG1 antibody	Acute myeloid leukemia & myelodysplastic syndrome	ASH 2021, Affimed Company.
	CTX-8573 (NKp30xBCMAxNK ADCC)	Symmetrical adapted IgG with C-terminal common light-chain appendage	Multiple myeloma	(83) Compass Therapeutics
	CTX-4419 (NKp30xBCMAxNK ADCC)	Symmetrical adapted IgG with C-terminal common light-chain appendage	Multiple myeloma	(84) Compass Therapeutics
	CYT-338 (Nkp46xCD38xNK ADCC)	Symmetrical adapted IgG (FLEX-NK)	Multiple Myeloma	(85) Cytovia Therapeutics Inc.
	CYT-303 (NKp46x GPC3xNK ADCC)	Symmetrical adapted IgG (FLEX-NK)	Hepatocellular carcinoma	(86) Cytovia Therapeutics Inc.
Multiple formats	NKG2Dx2B4	Available in 3 formats: 2 different symmetrical adapted IgG (with appendage at either N- or C-termini), 1 linked scFv	*Not available*	(87)
Nanoengager	CD16Ax4-1BBxEGFR	Nanoengager	EGFR^+^ cancers, e.g Lung cancer	(88)
Fused ligand peptides	OMCPxIL-2	Fusing OMCP peptide with IL-2 mutant with lower affinity for IL-2Rα	*Not available*	(89)
	ULBP2xBB4 (ULBP2-BB4)	Linked extracellular domain of receptor ligand	Multiple Myeloma	(90)
	TriNKET DF1001 (HER2 targeting)	Not disclosed	Breast cancer	Dragonfly Therapeutics Company

It is noteworthy that CD16a is rapidly cleaved from NK cells after activation by metalloproteases, such as ADAM17 (95, 96), or matrix metalloproteases (MMPs), like MMP25 (97). The downregulation of CD16a expression may facilitate NK cells to detach from their current target after cytolysis, enabling them to engage the next target cell (98). On the other hand, this process may also pose a disadvantage for CD16a-engaging NKCE development, as CD16a downregulation may make CD16a-targeted NKCEs less effective. The use of MMP inhibitors in combination with CD16a-targeting NKCEs could be a potential strategy to prevent CD16a shedding from NK cells and enhance their activation (99). Noteworthy, NKCEs that target CD16a can also bind to the membrane-bound CD16b receptors on neutrophils, whose extracellular domain is remarkably similar to that of CD16a on NK cells (100). In addition, soluble CD16 present in the serum, primarily consisting CD16b shed from the surface of neutrophils (101) and a smaller amount of CD16a shed from NK cells (96), creates a considerable “sink” effect. This effect could potentially compete with CD16a on NK cells for the binding of CD16a-engaging NKCEs, considerably diminishing their effectiveness.

#### 4.1.2 NKG2D

NKG2D is a disulfide-linked homodimeric C-type lectin-like receptor expressed in NK cells, NKT cells, a subset of CD8^+^ T cells, and a subset of γδ T cells (37). Although its intracellular domain lacks ITAMs and does not bind with an ITAM-bearing adaptor, NKG2D associates with an adaptor protein known as DAP10. DAP10 contains a tyrosine-based YxxM motif in its cytoplasmic tail. Upon tyrosine phosphorylation by Src-family kinases (102, 103), DAP10 induces the recruitment and activation of PI3K (104) and the Grb2/Vav1/PLCγ signalling complex (102) (**Table 1**).

The engagement of NKG2D activates the cytotoxic function and cytokine release of NK cells (105, 106). Notably, NKG2D-mediated tumor cell killing occurs more swiftly than that mediated by CD16a (98), although its activation strength is comparatively weaker. Interestingly, NKG2D-activated NK cells demonstrate a higher likelihood of sequential killing tumor cells compared to those activated by CD16a (98). Furthermore, NK cells are less motile in tumors lacking NKG2D ligands than those with NKG2D ligands (107), suggesting that targeted blockage of NKG2D ligands in tumors could help retain tumor-infiltrated NK cells within the tumor.

Several studies have evaluated the effectiveness of NKCEs that target NKG2D for treating multiple myeloma (MM) (**Table 2**). A linked single-chain variable fragment (scFv) NKCE, known as CS1xNKG2D, which targets CS1 (also called SLAMF7, a tumor antigen expressed on MM), has been demonstrated to induce dose-dependent cytotoxicity against CS1^+^ MM cells *in vitro*, stimulate IFN-γ production, and enhance the survival of NSG mice implanted with human MM cells (54). Moreover, various NKG2D^+^ cytotoxic immune cells, including NK, NKT, and CD8+ T cells, have been demonstrated to lyse MM cells additively when induced by the NKG2D-targeted NKCE (54).

Another NKCE that targets NKG2D, BCMAxMICA (also known as 2A9-MICA), utilizes the MHC I-related chain A (MICA), which is a ligand of NKG2D, to activate NKG2D on NK cells (67). 2A9-MICA is comprised of the extracellular region of human MICA and an scFv against BCMA, connected by a G_4_S linker (67). It has been reported that adding 2A9-MICA to a co-culture of NK-92 and MM cells increases NK-92 cell degranulation and enhances the cytolysis of BCMA^+^ MM cells by NK-92 cells. Additionally, 2A9-MICA could significantly suppress tumor growth in a nude mouse model without evident toxicity (67). However, it is noteworthy that several cancers exhibit high levels of soluble MICA (108), which could potentially compete with MICA-bearing NKCEs for binding of NKG2D, thereby reducing their effectiveness.

Bispecific antibodies targeting NKG2D and 2B4 receptors have been evaluated for cytotoxicity and IFNγ production of NK cells (87). These antibodies, designed in various formats, such as linked scFv, and symmetrical IgG with appendage at C- or N-terminus, have demonstrated a superior capability for activating NK cells compared to a combination of monospecific antibodies against NKG2D and 2B4, suggesting that co-engagement of these two receptors could synergize NK cell activation (87). It is thus intriguing to hypothesize that NKCEs, simultaneously engaging NKG2D, 2B4, and a tumor-associated antigen, could induce a more potent NK cell activation and more robust anti-tumor activity.

#### 4.1.3 Nkp30

Nkp30 is an Ig-like transmembrane protein with a single V-type extracellular domain (109). It is constitutively expressed in resting and activated NK cells (110). Similar to CD16a, Nkp30 is also associated with disulfide-linked homodimers or heterodimers of CD3ζ (109) and/or FcϵRIγ (111) chain for signal transduction (**Table 1**). As a natural cytotoxicity receptor (NCR) family member, Nkp30 triggers NK cell degranulation and cytokine release upon engagement (66, 111). Unlike CD16a, Nkp30 is not rapidly downregulated following NK activation, rendering it a more promising target over CD16a for better efficacy and persistence.

An NKCE incorporating the Nkp30-specific ligand, B7-H6, can adequately activate NK cells and direct them towards tumor cells (66), leading to the secretion of IFN-γ and TNF-α (112). Studies have shown that the cytolysis of HER2^+^ cells by NK cells, mediated by the Nkp30xHER2 NKCE, is comparable to or slightly superior to that mediated by therapeutic mAbs Trastuzumab and Cetuximab (66). Currently, two Nkp30xBCMA NKCEs, CTX-4419, and CTX-8573, are undergoing preclinical assessment for their effects in treating MM (83, 84). Both NKCEs were constructed using anti- Nkp30 and anti-BCMA antibodies that share a common light chain. The two anti-Nkp30 Fab fragments are appended at the C-terminus of the heavy chain of the anti-BCMA IgG1 antibody, which has an afucosylated Fc for enhanced binding with CD16a (**Table 2**). These NKCEs have been demonstrated to induce greater tumor cell killing and IFN-γ release than therapeutic mAbs, such as Elotuzumab or Daratumumab (83, 84). Notably, even without CD16a engagement by the Fc region, both NKCEs can still elicit the lysis of tumor cells via Nkp30 (83, 84). Both CTX-4419 and CTX-8573 activate NK cells in the presence of BCMA^+^ tumor cells, indicating good safety due to minimal tonic activation (83, 84).

However, the lower cell surface expression of Nkp30 might be a concern when considering it as a target for NCKE development. A study has shown that each NK cell has approximately 1000 Nkp30 molecules expressed on the cell surface, which is considerably lower than the density of CD16a (~70,000 molecules per NK cell) (66). Therefore, designing Nkp30-targeted NKCEs to accommodate the larger spacing between antigens might necessitate a more flexible structure.

#### 4.1.4 Nkp46

Nkp46 is an NCR expressed on all mature NK cells and is associated with CD3ζ and/or FcϵRIγ homodimers or heterodimers for signal transduction (113). Despite sharing many characteristics with Nkp30, Nkp46 has two extracellular C-type Ig domains (114), whereas Nkp30 has only one Ig domain (**Table 1**). Engagement of Nkp46 alone only weakly activates resting NK cells (115), but co-engagement with other activating receptors, such as 2B4, DNAM1, or CD2, can significantly enhance NK cell activation (115). Notably, unlike CD16a, Nkp46 is not downregulated in the tumor-infiltrated NK cells in several cancers, including lung carcinoma (116), acute myeloid leukaemia (117), and breast cancer (118). Therefore, NKP46 holds promise as a target for NKCE development.

A Nkp46xTAA NKCE is constructed using a single-armed adapted IgG format, targeting Nkp46 by the Fab at the C-terminus and the tumor antigen at the N-terminus (79) (**Figure 2**). Besides activating NK cells by engaging Nkp46, this NKCE also promotes NK cell ADCC through its Fc region via CD16a binding. It has demonstrated better suppression of CD19^+^ or CD20^+^ tumor cell growth compared to the therapeutic mAb Rituximab or Obinutuzumab in both *in vitro* (80) and *in vivo* studies (79).

Another tetravalent NKCE, Nkp46xCD38 (CYT-338), is also undergoing development. CYT-338 has an IgG1 backbone that can mediate NK cell ADCC through its Fc region. It recognizes Nkp46 through its Fab regions; additional Fab fragments targeting the tumor antigen are symmetrically appended at the N-terminus of heavy chains of the IgG backbone (FLEX-NK, **Figure 2**). Mutations have been introduced at the IgG backbone’s constant regions (CH1 and CL) to minimize the mispairing of heavy and light chains and facilitate the proper chain pairing. CYT-338 has shown a 3-fold higher binding to MM cell lines than Daratumumab and has demonstrated greater dose-dependent NK cell cytolysis and cytokine production (119). Notably, these NKCEs may link two NK cells together by co-engaging Nkp46 and CD16a or Nkp46 and CD38 on NK cells. Thus, it is yet to be determined if there is a risk of fratricide of two neighbouring NK cells that could potentially be co-engaged by the same NKCE. This is especially pertinent given that the monospecific, bivalent therapeutic mAb Dratumumab, which targets CD38 on NK cells, has been reported to induce NK cell fratricide (120).

#### 4.1.5 Nkp80

Nkp80 (KLRF1) is a homodimeric C-type lectin-like receptor similar to NKG2D (121). It is primarily expressed in NK cells (122) (**Table 1**). The cytoplasmic tail of Nkp80 bears a hemi-ITAM-like sequence (YxxL), which, upon binding, associates with Lck and Syk kinases (123). The only known natural ligand for Nkp80 is the activation-induced C-type lectin (AICL) (121).

An Nkp80-targeting NKCE, known as AICLxHER2-scFv, has been developed using AICL to engage Nkp80 (66). This NKCE contains the extracellular domain of AICL and the scFv of an anti-HER2 antibody. AICLxHER2-scFv has demonstrated its capability to activate NK cells and direct NK cytolysis towards HER2^+^ tumor cells, exhibiting synergistic effects with therapeutic mAb Trastuzumab or Cetuximab (66). Interestingly, it has been suggested that simultaneous Nkp80 and DNAM-1 activation could significantly enhance NK activation, whereas Nkp80 does not show synergy with Nkp30 (66). These findings highlight Nkp80 as a promising target for NKCE development. However, the relatively low surface expression Nkp80 (about 5,000 copies per NK cell) (66) may require some special NKCE design considerations such as enhanced flexibility and multivalency to improve the efficacy of Nkp80-targeted NKCE.

#### 4.1.6 NKG2C

NKG2C, also known as CD159a, is an activating receptor that specifically recognizes HLA-E (124). It forms a heterodimer with CD94 and is associated with DAP-12 through a positively charged residue in its transmembrane domain (125). (**Table 1**) NKG2C is predominantly expressed in mature NK cells that lack NKG2A, and its increased expression signifies a “memory” NK cell phenotype (126).

Similar to CD16a, NKG2C can trigger strong activation of NK cells even without co-activation (127). However, in contrast to CD16a, NKG2C does not rapidly shed off post-NK cell activation, making it a promising candidate for NKCE development. An NKCE, known as NKG2CxIL-15xCD33, comprising an anti-NKG2C scFv and an anti-CD33 scFv, linked by a wild-type IL-15 in the middle (62), has demonstrated satisfactory NK cell activation, expansion, and cytotoxicity against CD33^+^ myeloid leukaemia cells both *in vitro* and *in vivo* in an NSG mouse model (62). Its efficacy in suppressing tumor growth suppression is comparable to 161533 NKCE (62).

However, one potential limitation of NKG2C compared to CD16a is its lower and variable expression level on the surface of NK cells (62, 128). The population of NKG2C^+^ NK cells correlates with an individual’s history of human cytomegalovirus (CMV) exposure. Individuals with previous CMV exposure will likely have a larger NKG2C^+^ NK cell population than those who are naïve to the virus (62). Even among CMV-seropositive individuals, the frequency of NKG2C^+^ NK cells could vary considerably (128, 129). Therefore, NKG2C-NKCE may not be effective for some patients due to a low population of NKG2C^+^ NK cells.

### 4.2 Engaging other potential activating receptors for NKCE development

Unlike most of the killer cell Ig-like receptor (KIR) family members, which are inhibitory, KIR2DS and KIR3DS are two activating receptors of the KIR family (130, 131). Despite sharing two or three Ig-like extracellular domains with other inhibitory KIRs, KIR2DS and KIR3DS possess a short cytoplasmic tail without ITIMs. Instead, they associate with ITAM-bearing adaptor DAP12 (132) (**Table 1**). Studies have shown that KIR2DS expression in NK cells is positively correlated with improved NK activation and cytokine production (133). Therefore, KIR2DS and KIR3DS are potential candidates for NKCE development. However, the high homology in the extracellular domains of the activating and inhibitory KIRs might make it challenging to generate specific antibodies or CDR sequences that can distinguish these two types of KIRs (131).

CD160 is an Ig-like glycosyl phosphatidyl inositol (GPI)-anchored transmembrane protein, similar to KIR receptors. It is expressed in a fraction of NK cells with high cytotoxicity and a subset of T cells (134) (**Table 1**). Upon engagement by its ligand HLA-C, CD160 can trigger robust NK cell activation leading to efficient tumor cell lysis (135) and secretion of TNF-α, IFN-γ, and IL-6 (136, 137), making it a potential target for NKCE development. However, CD160 has been shown to undergo downregulation following NK cell activation by phorbol ester (138).

DNAM-1, also known as CD226, is another activating receptor constitutively expressed in NK cells, T cells, and certain myeloid cells. It is a transmembrane glycoprotein with two Ig-like domains (139, 140) (**Table 1**). Unlike other Ig superfamily members, the cytoplasmic tail of DNAM-1 contains four tyrosine residues (Y293, Y300, Y322, and Y325), one asparagine residue (N324) and one serine residue (S329), which can recruit Src family kinase Fyn, adaptor Grb2 and PKC upon phosphorylation (140). Although engagement of DNAM-1 alone does not increase NK cell cytotoxicity, it promotes NK cell adhesion to the target cell and granule polarization (140) and can synergize with 2B4 to enhance NK cell cytotoxicity (47). DNAM-1 competes with inhibitory receptor TIGIT and CD96 for binding with their shared ligands PVR (CD155) and Nectin-2 (CD112) (141). A study showed that a DNAM-1-engaged NKCE, PVRxHER2-scFv, failed to display potent cytotoxicity, probably due to PVR’s competitive binding with inhibitory receptors (66). Moreover, DNAM-1 has a low surface expression on NK cells. Therefore, future DNAM-1-engaged NKCE development requires more specific design strategies.

2B4 (SLAMF4, CD244) belongs to SLAM (Signaling Lymphocytic Activation Molecule) family and is expressed in NK and CD8+ T cells (142). It has two Ig-like extracellular domains and a cytoplasmic tail with four immunoreceptor tyrosine-based switch motifs (ITSMs), which can recruit SAP and protein tyrosine kinase Fyn, and SHP1/2 (143, 144). Although engagement of 2B4 alone does not activate NK cells, it can synergize with Nkp46, NKG2D, and DNAM-1 to achieve significantly enhanced NK cell activation (47). Therefore, 2B4 is frequently employed as an auxiliary receptor in the design of some multispecific NKCEs.

### 4.3 Incorporating stimulatory cytokine into NKCEs

Stimulatory cytokines can dramatically enhance NK cell proliferation and cytotoxicity. For example, the anti-tumor capabilities of functionally impaired tumor-infiltrated NK cells can be fully restored with interleukin (IL)-2 and IL-15 (145, 146). A clinical trial also revealed that IL-15 infusion in cancer patients could significantly expand NK and memory CD8^+^ T cell populations (147). These findings suggest that incorporating stimulatory cytokines, such as IL-2 or IL-15, into NKCEs may synergistically enhance their antitumor potency.

The incorporation of an IL-15 moiety into an NKCE that has two scFv-segments, with one binding CD16a on NK cells and the other one binding tumor-associated antigens on tumor cells, can significantly enhance the cytotoxicity of NK cells and their pro-inflammatory cytokine production compared to NKCE without IL-15 (38, 59, 60) (**Table 2** and **Figure 2**). An IL-15-incorporated NKCE against acute myeloid leukaemia (AML), referred to as CLEC12A TriKE or CD16axIL-15xCLEC12A (**Table 2**), has been shown to specifically expand NK cells, but not T cells, leading to the potent killing of AML cells by NK cells (61). Another study showed that IL-15-incorporated NKCE (161533 TriKE/GTB-3550) rectified the functional defects of NK cells and extended the survival of cancer patients post-hematopoietic stem cell transplantation (38). GTB-3550 has shown promise in treating CD33^+^ malignancies (NCT03214666). These findings demonstrate the significant clinical values of incorporating IL-15 into NK cell therapy for cancer treatment.

Similar to IL-15, IL-2 is also a potential stimulator for NKCE. However, the unspecific activation of Treg cells by IL-2 (148) can be problematic. The activity of IL-2 can be limited to NK cells by incorporating it into an NKCE but negating its effect on Treg cells, which can suppress many immune cells, including NK cells (149). A fusion protein consisting of NKG2D ligand OMCP and a modified IL-2 has shown the feasibility of this restricted delivery of IL-2, resulting in specific activation of NKG2D+ cytotoxic cells (89).

Additionally, combining IL-15, IL-12, and IL-18 can induce memory-like NK phenotype, leading to extended persistence and superior cytotoxicity of NK cells (150). Hence, it would be interesting to investigate if NKCEs incorporating IL-15, IL-12, and IL-18 can induce more potent NK cell cytotoxicity than NKCEs without cytokine incorporation.

### 4.4 Integrating immune checkpoint receptor blockade into NKCEs

#### 4.4.1 Blockade of PD-(L)1

Although NKCEs that engage activating receptors can facilitate the formation of immune synapses between healthy NK and tumor cells, leading to NK cell activation and tumor cell lysis, the immunosuppressive tumor microenvironment (TME) can dampen the expression of these activating receptors on the NK cell surface (151, 152). This may weaken the engagement of tumor-infiltrated NK cells by NKCEs, resulting in reduced NK cell activation and expansion. On the other hand, tumors and other cells in the TME upregulate the expression of ligands for NK cell inhibitory receptors, which is a mechanism that inhibits the antitumor function of NK cells. Preventing the engagement of these inhibitory receptors on NK cells by their ligands can potentiate the antitumor function of NKCEs. For instance, EGFRxCD16axPD-L1 is a trispecific NKCE that activates NK cell-induced killing of EGFR^+^ tumor cells through CD16a while blocking the binding of PD-L1 to the immune checkpoint receptor PD-1 on activated NK cells, thus reducing NK cell exhaustion (81). Moreover, the anti-PD-L1 arm on NKCE provides additional recognition of tumor cells, as tumor cells often express PD-L1 (153). Studies have demonstrated that the trispecific EGFRxCD16axPD-L1 NKCE exhibits superior antitumor potency compared to the bispecific EGFRxCD16a NKCE or anti-EGFR mAb (81).

However, one potential issue that may arise when integrating checkpoint blockage into NKCEs concerns dosing. Optimal effects from immune checkpoint blockade typically require high dosage levels (154). However, such high dosages could potentially cause severe toxicity, especially considering that the NK-activating component of the NKCE is typically designed to function effectively at low concentrations.

#### 4.4.2 Blockade of other inhibitory immune checkpoints

Several other inhibitory receptors are also known to suppress NK cell antitumor function, particularly within the TME of various cancers. These receptors include NKG2A, TIGIT, TIM3, CD96, and KIR2DL/KIR3DL, in addition to PD-1/PD-L1. NKG2A can suppress NK cell function by recruiting SHP-1/2 through its cytoplasmic tail’s ITIMs upon the engagement by non-classical class I MHC molecules HLA-E (155). Monalizumab, an NKG2A-blocking IgG4 mAb, has been shown to boost NK cell activation and synergistically control tumor size with limited toxicity when combined with a PD-L1 blocking mAb in Phase 2 clinical trial (156). A blocking antibody against TIGIT, an inhibitory receptor on NK cells bearing ITIM- and Ig tail-tyrosine (ITT)-like motif, can reverse the exhausted phenotype of TIGIT-upregulated tumor-infiltrated NK cells, constrain tumor growth, and improve the survival tumor-bearing mice *in vivo* (157, 158). TIM-3, another NK exhaustion receptor, inhibits NK cell function (159, 160). TIM-3 blockade enhances NK cell cytotoxicity against MM cells (161) and cells chronically infected by the hepatitis B virus (162). Moreover, another mAb, IPH4102, which blocks inhibitory KIR3DL2, has demonstrated promising clinical activity in patients with cutaneous T-cell lymphoma (163). CD96 is another inhibitory receptor, also described as TACTILE (164). Human CD96^+^ NK cells demonstrate an exhausted phenotype with reduced IFN-γ, TNF-α, perforin, and granzyme B expression, and blocking CD96 interaction with its ligands enhances the cytotoxicity of NK cells (165, 166). NK cells in tumor tissue show a higher level of CD96 than those in the peritumoral region (165). Moreover, the combinatory blockade of PD-1 and CD96 has significantly suppressed tumor growth compared to blocking PD-1 alone (167). Therefore, blocking CD96 can be a novel strategy for reviving NK cells from exhaustion.

It is important to note that the blockade of immune checkpoints may not be effective for all types of cancers. For example, a study investigating the blocking of PD-1 and/or TIM-3 in early chronic lymphocytic leukemia patients failed to demonstrate any significant effect (168). Therefore, the effectiveness of immune checkpoint blockade therapy is ultimately contingent on the unique biology of each specific cancer disease.

## 5 Developing NKCEs with desirable functionality and manufacturability

Engaging the appropriate target receptors is crucial, as it determines the strength and persistence of NK cell activation and the subset of NK cells that get activated. Therefore, substantial efforts are being devoted to selecting more suitable target receptors for NKCE development. However, the ultimate goal of NKCE development for cancer treatment is to generate potent NKCEs with desirable functionality and manufacturability, both of which are also significantly impacted by their molecular structures. In the following sections, we will discuss several critical aspects that influence the functionality and manufacturability of NKCEs.

### 5.1 Formation of an optimal cytolytic immune synapse

The formation of a cytolytic immune synapse (IS) between NK and cancer cells is a prerequisite for an NKCE to eradicate cancer cells effectively. The IS formation involves three phases: the recognition and activation phase, the effector/lysis phase, and finally, the detachment or termination phase (107, 169). The crucial role of NKCEs is facilitating IS formation during the early recognition and activation phase by bridging the interaction between NK and target cells. This interaction subsequently triggers NK cell activation through receptor engagement, resulting in cancer cell lysis (169).

Efficient clustering of receptors on both NK and target cells during their early stages of interaction is crucial for IS formation and NK cell activation (170). Enhancing the valency of a monospecific antibody has been shown to potentially improve the target binding by increasing its avidity (171, 172). This principle is equally applicable to bispecific antibodies. For example, the CD20-targeted T-cell bispecific antibody (RG6026, Roche), which has two CD20-binding Fabs and one CD3-binding Fab, has demonstrated superior potency than a similar format with only one CD20-binding Fab (173). Similarly, the loss in binding and killing ability in the T-cell bispecific antibody with low-affinity variants of HER2-binding Fab can be rectified by the bivalent engagement of two low-affinity Fab variants (174). It is noteworthy that receptor density also impacts the avidity-mediated binding of antibodies. Downregulation of some tumor cell surface antigens is a prevalent mechanism for immune escape (175), such as the downregulation of BCMA in MM cells (176). The cell surface BCMA antigens shed from MM cells and become soluble antigens, which might even compete with those membrane-bound BCMA antigens to bind BCMA-targeted NKCEs and reduce their efficacies. Therefore, the efficient interaction of NKCEs with NK and target cells is critical for developing NKCEs with high efficacy.

Spatial constraint in the synaptic cleft is another critical factor for optimal NKCE-meditated IS formation between NK and target cells. The synaptic cleft of most physiological IS is approximately 10-30 nm in size (177). Molecules exceeding this size could be excluded from the synaptic cleft and unable to induce IS formation (178). Thus, there is a size limit for NKCE molecules. Monoclonal antibodies, typically about 10 nm in diameter (179, 180), fit nicely into the synaptic cleft, thereby promoting stable IS formation. However, the addition of antibody moieties in NKCEs, necessary for achieving multispecificity, or increased valency, inevitably increases their size and compromises their ability to mediate stable IS formation due to the synapse structure distortion. Replacing larger antibody fragments with smaller ones, such as scFv or nanobody, may overcome the size constraint. For example, the ability of a NKG2Dx2B4 NKCE to activate NK cells has been assessed in different formats, including modified IgG format and linked scFv (87). The NKG2Dx2B4 NKCE, formatted as an scFv and smaller size than the IgG format, exhibited comparable efficacy of inducing IFN-γ and granzyme B secretion, with only a marginal lesser capacity to induce NK cell degranulation (87). These findings indicate that it is feasible to maintain full functionality even when larger antibody moieties are replaced with smaller ones in the design of NKCEs.

Furthermore, the physical protrusion and conformation of the tumor antigens and NK cell receptors in the extracellular region could affect the design of NKCEs due to the spatial constraints of the IS. The NKCE’s appropriate size is crucial for stable IS formation, bridging the ectodomains of both NK receptors and tumor antigens. For example, CD20 is less protruding from the cell surface compared to CD19 (181, 182), suggesting that different designs may be required when constructing NKCEs that engage with CD19 or CD20, even though both antigens are expressed on the same B cells and the NKCEs target the same NK cells.

### 5.2 Format design for NKCE development

To date, various NKCE formats have been evaluated for their functionality and manufacturability, with many adapted from the formats used for T cell engagers. Based on their molecular structure design, NKCE formats can be broadly classified into two major categories: fragmented formats and adapted IgG formats. The fragmented formats generally have smaller sizes than the adapted IgG formats due to the absence of the Fc region (**Figure 2**).

#### 5.2.1 Fragmented formats

Most of the fragmented formats are derived from the variable (V) region of the heavy (H) and light (L) chains of IgGs and lack the constant regions (**Figure 2**). The linker between different V regions is the most common component of these fragmented formats. This linker connects two V regions, typically a pair of VH and VL, to form an scFv and construct the NKCE. The linker’s length between the two V regions determines their pairing (183). Linkers shorter than 10 amino acids do not allow the V regions to rotate and fold, leading to inter-chain pairing, as seen in the diabody (**Figure 2**). Studies suggest that the scFv’s linker should span at least 3.5 nm to permit the two V regions to fold and pair to form scFv (184). A typical linker is flexible and hydrophilic, minimizing structural interferences during protein folding within the cell. Several linker options are available to bridge the V regions, such as (G_4_S)_n_/(G_3_S)_n_ linker (185), 218s linker (GSTSGSGKPGSGEGSTKG) (186), or HMA linker (PSGQAGAAASESLFVSNHAY) (49) (**Table 3**). The (G_4_S)_n_ linker is most commonly used (187, 188), due to its high flexibility, low immunogenicity, and serine residues’ contribution to solubility. Moreover, the 218s linker is reported to be more proteolytic stable and has reduced aggregation (186), while the HMA linker exhibits lower immunogenicity (189). In addition to these conventional linkers, other linker designs are also possible. Phage display can tailor the linker sequence for a specific antibody, especially when conventional linkers do not work well (190).

**Table 3:** Commonly used linkers in NKCE design.

Linker	Amino Acid Sequence	Features
(G_4_S/G_3_S)_n_ linker	(GGGGS)_n_ or (GGGS)_n_	* Conventionally used, length adjustable; * High flexibility, low immunogenicity, and good solubility
218s linker	GSTSGSGKPGSGEGSTKG	* Proteolytic stable * Reduced scFv aggregation
HMA linker	PSGQAGAAASESLFVSNHAY	* Low immunogenicity

Secondary bonds, like disulfide bonds, are critical to successfully assembling fragmented-type NKCEs. For example, in the tribody format (Fab construct, see **Figure 2**), the disulfide bond between the heavy and light chains facilitates the pairing. Additionally, introducing a disulfide bond between intra-domain regions can help stabilize the structure of engineered IgG antibodies, particularly when the domain structure lacks intrinsic stability (191).

#### 5.2.2 Adapted IgG formats

Adapted IgG formats, which retain the antibody’s Fc region, are larger than fragmented formats (**Figure 2**). Typical engineering methods of this type of NKCE involve changing the specificity of one arm of the IgG backbone and/or appending an antigen-recognizing moiety, either scFv or Fab, to the N- or C-terminus of the original IgG (**Figure 2**). Depending on the position of the modifications, adapted IgG formats could be further subdivided into asymmetrical and symmetrical types (**Figure 2**).

Compared to the relatively simple folding requirement of the fragmented format of NKCEs, asymmetrical adapted IgG NKCEs present more challenges during protein folding. The main issues are the unsought homodimerization of heavy chains and the mispairing of the heavy and light chains of asymmetric IgG NKCEs when different NKCE parts are produced in the same cell (**Figure 2**). Previously, the production of those asymmetric IgGs through the hybrid hybridomas has encountered low yield due to the undesired homodimers of the heavy chains and mispaired light chains with non-cognate heavy chains (192).

To date, various techniques have been developed to overcome these problems. For example, chimeric rat/mouse-derived quadroma (193), knobs-into-holes (194), strand-exchange engineered domain (SEED) (195), and heterodimerization/electrostatic steering interaction (196) methods are used to enhance the heterodimerization of the heavy chains. CrossMab (197), CH1/CL interface engineering, or IgG/TCR chimaera (198) approaches are employed to minimize the mispairing of heavy and light chains. These technologies create and utilize a higher affinity for one chain to its desired pairing chain over other chains, thereby increasing the yield of NKCEs with the correct chain pairing. Another potential solution to the issue of the mispairing of heavy and light chains could involve using a common light chain. However, this strategy might not be applicable to all the antibodies, particularly those where the CDR sequences of both heavy and light chains are equally critical for determining the antigen-binding specificity.

Alternatively, instead of producing different parts of an NKCE in one single cell, these parts could be synthesized separately and subsequently assembled to form the functional format. This approach, known as co-culture (199), involves producing two distinct half-antibodies in two separate cell lines and combining them to generate the complete structure in a 1:1 molar ratio. While the co-culture method can yield substantial protein quantities, it may escalate costs and contamination risks due to the need for additional purification steps (199).

Due to their symmetrical structures, the symmetrical IgG formats can easily circumvent the chain pairing issues inherent to the asymmetrical formats, potentially reducing production costs. However, a concern for the symmetrical IgG formats is their considerably larger size than the asymmetrical formats when achieving the same multispecificity. The increased size could theoretically compromise the yield and increase the immunogenicity of the symmetrical NKCEs (200, 201).

It is noteworthy that the choice of the antigen recognition moiety can significantly impact the function of NKCEs. Although the Fab format for antigen recognition poses a challenge in correctly pairing heavy and light chains, it is functionally more potent than the scFv format. A study has shown that certain scFvs, which failed to function *in vivo*, could operate once switched to the Fab format (202).

#### 5.2.3 Other non-antibody-based formats

Some unconventional formats, which are not purely antibody-based, can also be employed in NKCE development (**Figure 2**). One such format involves fusing the ligands of the corresponding receptors on both the tumor and NK cells to engage the NK cell with the tumor cell (90). This strategy offers a broader range of receptor targets for NKCE engineering; however, it does not prevent competition with the endogenous ligand binding to the receptors. Another approach, known as nano-engager, utilizes the ability of nanoparticles to be coated with multiple antibodies (88) (**Figure 2**). This method allows a single nanoparticle to display multiple specificities, directing NK cells towards tumor cells.

Nanobodies, also known as VHH (Variable Heavy domain of Heavy chain) antibodies, present a promising alternative for antigen recognition in NKCEs. Unlike scFv or Fab, which is constructed using VH and VL, nanobodies are camelid single-domain antibodies that can achieve specific antigen-binding using heavy chains only (203). Therefore, nanobodies can circumvent the pairing issue of the Fab format and the folding issue of scFv, making them a promising candidate in future NKCE designs. The feasibility of incorporating nanobody into NKCE has been demonstrated by the CD16axIL-15xCLEC12A NKCE (61) (**Table 2**). In this NKCE, a humanized anti-CD16a VHH fragment, instead of the conventional anti-CD16a scFv, was incorporated into the NKCE and could mediate efficient NK cell activation and tumor cell lysis (61).

Recently, a new form of antigen recognition called affibody has also emerged due to advancements in protein engineering technologies (204). Affibodies are constructed using three-helix subdomains, approximately 7 kDa in size (205). Randomization of the amino acids on two of the helices has been found to generate a large library, from which some potent antigen binders to the antigen can be isolated (205–208). For instance, a CD16axBCMA NKCE developed using affibodies has illustrated the potential of this new format to replace the conventional scFv moieties in the design of NKCEs (**Table 2**). This compact NKCE (15-23 kDa), consisting of CD16a- and BCMA-specific affibodies connected by a linker, has demonstrated potent efficacy in activating primary NK cells, initiating synapse formation, and specifically lysing MM cells (58).

### 5.3 Other factors affecting NKCEs’ functionality

In addition to the direct effect of format design on the functionality of an NKCE, other factors, such as tissue infiltration and persistence, should also be considered during the development of NKCEs. These factors can significantly influence the therapeutical efficacy of NKCEs.

Currently, most NKCEs undergoing preclinical testing are of fragmented formats (**Table 2**). Their smaller size (50-100 kDa) affords them excellent tissue infiltration properties and substantially lower immunogenicities. However, these small-size NKCEs often lack *in vivo* persistence due to renal clearance and degradation (209, 210), requiring frequent infusions to the body to maintain effective drug levels. This will increase treatment costs due to the recurrent patient hospital visit for repeat infusions. Thus, strategies to extend the *in vivo* lifespan of these small-size NKCEs have been proposed. One such approach is to bind or fuse them to long-lived serum proteins, like albumin (half-life 2-4 weeks) (211, 212), or conjugate them to the Fc region of IgG (212, 213). Additionally, attaching the small-size protein to a chemical polyethene glycol (PEG), which could also prolong its half-life (209), might be feasible to extend the half-life of the smaller NKCEs. Several approved protein drugs, such as interferon-α 2b (IFN-α 2b), granulocyte-colony-stimulating factor (G-CSF), or human growth hormone (hGH), have used this method for longer persistence (214). Another strategy involves multimerizing NKCEs in fragmented format to increase their size. For instance, AFM13, a tetravalent bispecific NKCE that binds to CD16a and CD30 on NK and tumor cells, is achieved by dimerizing two V domains. In a Phase 2 trial, AFM13 demonstrated promising results for treating multi-refractory Hodgkin Lymphoma patients (91, 215).

As mentioned earlier, adapted IgG formats typically have a larger size, usually exceeding 150 kDa (**Figure 2**). While their larger size may present challenges in tissue penetration, the inclusion of the Fc region significantly enhances their *in vivo* persistence (216). Additionally, the Fc region could recruit mechanisms such as ADCC, complement-dependent cytotoxicity (CDC), and antibody-dependent cellular phagocytosis (ADCP) in immune cells other than NK cells, adding a bonus to tumor cell killing (217). The most common approach to achieve multispecificity of the adapted IgG format is to add appendages with different antigen specificities to the C- or N-terminus of the IgG protein (81, 193, 218–222). (**Figure 2**) Nevertheless, incorporating appendages into NKCEs would inevitably increase their size, making tissue penetration more challenging and potentially enhancing immunogenicity (223, 224). Hence, balancing multispecificity, desired tissue penetration, and low immunogenicity is critical for developing NKCEs in the adapted IgG format to achieve satisfactory therapeutical efficacy.

### 5.4 Manufacturing of NKCEs

The choice of the production system of NKCE is also an essential factor in their development, as it determines the yield of NKCEs and the necessity for further downstream processing. Generally, two host systems are most commonly employed: bacterial and mammalian systems (refer to **Table 4** for specific systems used for individual NKCE). The bacterial system is frequently used for producing fragmented formats, such as linked-scFv type NKCEs, although some are also produced using the mammalian system, such as HEK 293 cells. On the other hand, adapted IgG formats are primarily produced in mammalian cells, such as CHO cells, due to the need for post-translational processing for successful chain pairing.

**Table 4:** Host systems for NKCE production.

Host system	NKCE name	Format	Reference
Bacteria	CD16axEpCAM	Linked scFv	(49)
	CD16axCD33	Linked scFv	(50, 51)
	CD16axCD19	Linked scFv	(52)
	CD16axCD19xCD22	Linked scFv	(52)
	CD16axCD133	Linked scFv	(53)
	1615133 TriKE (CD16axIL-15xCD133)	Linked scFv fused with cytokine	(59)
	CD16axIL-15xEpCAMxCD133	Linked scFv fused with cytokine	(60)
	161519 (CD16axIL-15xCD19)	Linked scFv fused with cytokine	(63, 64)
	GTB-3550/161533 TriKE (CD16axIL-15xCD33)	Linked scFv fused with cytokine	(38, 65) GT Biopharma
	CD16axEGFR	Diabody	(75)
	CD16axIL-15xHER2 (CAM1615HER2)	Linked VHH fused with scFv	(56)
	CD16axIL-15xB7H3 (CAM1615B7H3)	Linked VHH fused with scFv	(57)
CHO cells	AFM13 (CD16axCD30)	Tand Ab	(40, 68, 69)
	CTX-8573 (NKp30xBCMAxNK ADCC)	Symmetrical adapted IgG with common light-chain appendage	(83)
	CD16axNkp46xTumor Ag.	Single-armed IgG-adapted	(79)
CHO-S cell line	NKG2DxCS1	Linked scFv	(54)
CHO-ZEN cells	CYT-338 (Nkp46xCD38x NK ADCC)	Symmetrical adapted IgG (FLEX-NK)	(85) Cytovia Therapeutics Inc.
Flp-In CHO cells	AFM22 (CD16ax EGFRvIII)	Tand Ab	Affimed
	AFM24 (CD16axEGFR)	Tand Ab. (AFM24_T) and Symmetrical adapted IgG1 antibody with C-terminal appendage (AFM24_I)	AACR 2020 Jun 22-24 Poster 5659 – AFM24, Affimed Company (93);; NCT04259450
Expi 293 cells	B7-H6xEGFR	Asymmetrical adapted IgG with Fab replaced by Ig-like V-type domain of the extracellular region of B7-H6	(82)
	NKG2CxIL-15XCD33	Linked scFv fused with cytokine	(62)
	CD16axNkp46xTumor Ag.	Single-armed IgG-adapted	(79)
	NKp46xCD19/20	Single-armed IgG-adapted	(80)
	NKp30xCD16axCD19/20	Single-armed IgG-adapted	(80)
	CD16axEGFRxPD-1	Asymmetrical adapted IgG	(81)
	CD16axIL-15xCLEC12A (CLEC12A TriKE)	sdAb (VHH α-CD16a) & scFv (α-CLEC12A) plus cytokine motif	(61)
FreeStyle 293F cells	NKG2DxHER2	Bispecific (bsFab) or bivalent Fab-like (bvFab) antibodies with VHH	(78)
	NKG2DxFMDV	Bispecific (bsFab) or bivalent Fab-like (bvFab) antibodies with VHH	(78)
	OMCPxIL-2	Peptide fused with IL-2 mutant with lower affinity for IL-2Rα	(89)
HEK 293 cells	2A9-MICA (MICAxBCMA)	Ligand peptide fused with scFv	(67)
	NKG2Dx2B4	Available in 3 formats: 2 different symmetrical adapted IgG (with appendage at either N- or C-termini), 1 linked scFv	(87)
	CD16axEGFR	Linked VHH	(55)
HEK 293T & COS cells	ULBP2xBB4 (ULBP2-BB4)	Linked extracellular domain of the receptor and the ligand	(90)
Lenti-X 293T cells	AICLxHER2	Extracellular domain of the ligand fused to HER2-scFv	(66)
	B7-H6xHER2	Extracellular domain of the ligand fused to HER2-scFv	(66)
	ULBP2xHER2	Extracellular domain of the ligand fused to HER2-scFv	(66)
	PVRxHER2	Extracellular domain of the ligand fused to HER2-scFv	(66)
	(HER2)_2_xCD16a	Tribody	(74)
Non-cell system	CD16ax4-1BBxEGFR	Nanoengager	(88)

The bacterial system allows inexpensive, rapid, and high-volume production of NKCEs, but proteins produced often require refolding (225). Typically, NKCE genes are inserted into an expression plasmid vector with an inducible promoter. When the bacteria culture reaches high density, the NKCE expression is induced. The NKCE proteins produced are isolated from the bacteria’s inclusion body. Harvesting raw NKCE proteins from the bacteria system usually take approximately 1-2 days (49), followed by another 2-3 days for refolding and purification (49).

On the other hand, expressing NKCEs in mammalian cell systems, such as HEK 293T or CHO cells, can bypass the refolding process as these cells can secrete fully functional NKCEs, which can be isolated from the supernatant. However, the yield from the mammalian cell system is typically lower than that of the bacterial system (54, 55). Usually, mammalian cells are co-transfected or transduced with expression vectors encoding the light or heavy chains. After around 6 days of culture, the supernatants are collected for the purification of NKCEs, followed by analyses of chain size and pairing (79). Although HEK 293T cells can offer post-translational modifications closest to those found in the human body (226), they are not as effective as CHO cells for large-scale production due to limitations in growth capacity, yield, and doubling time (227).

## 6 Conclusion

**Figure 3:**
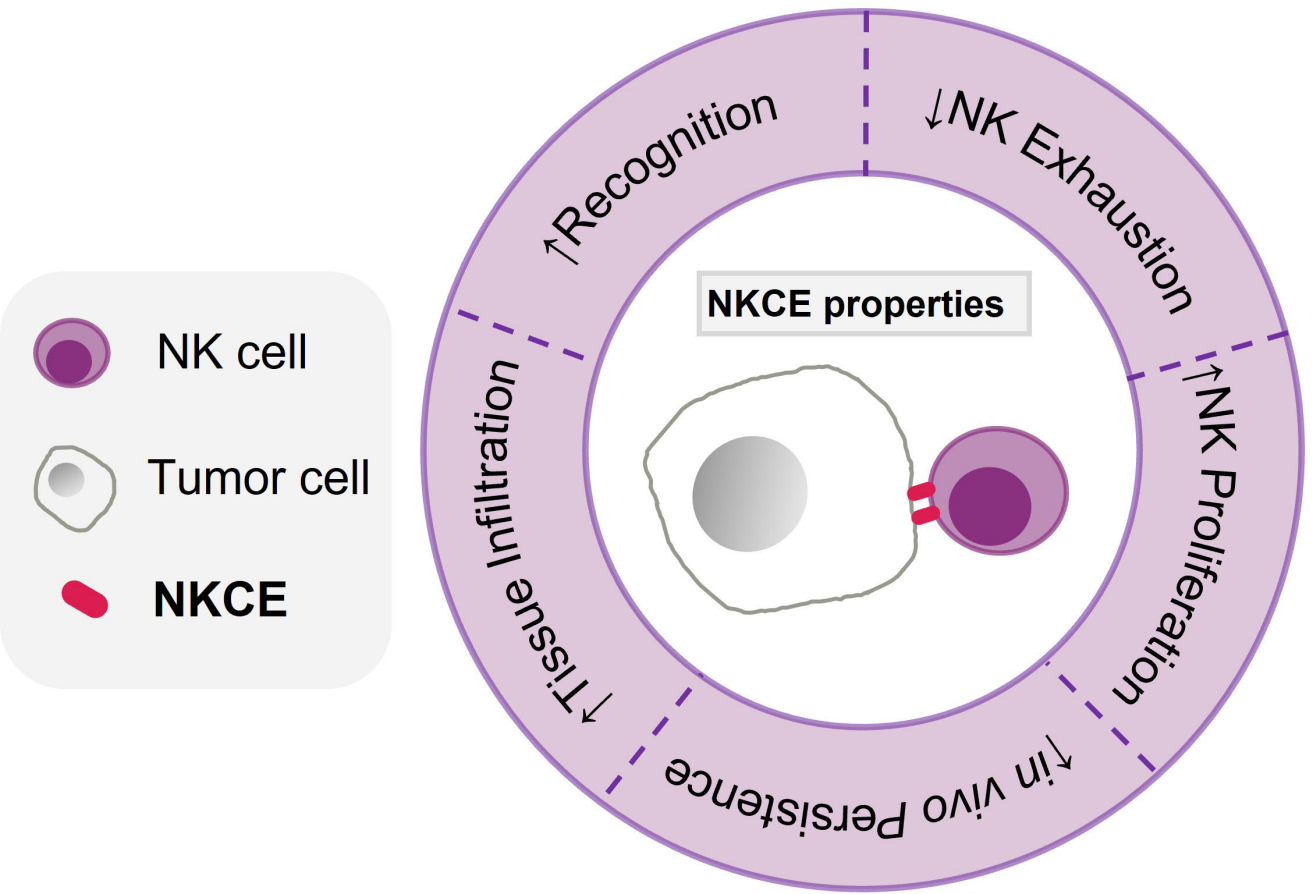
Characteristics of an effective NKCE. The characteristics of an effective NKCE include high specificity and selectivitity to tumor antigen (Recognition), being able to infiltrate into the tumor tissue effectively, enhance NK proliferation (within tumor), reduce NK exhaustion in TME, and persist *in vivo* for long period.

NKCEs have exhibited considerable potential as a new immunotherapeutic modality for cancer treatment. While the majority of current NKCEs targets CD16a and NKG2D, other activating receptors also display promising therapeutic potential and deserve further exploration. Moreover, a strategy that combines triggering activating receptors with blocking inhibitory receptors presents an effective approach to achieving enhanced NK cell activation. The inclusion of stimulatory cytokines in NKCEs has been shown to improve their therapeutical efficacies in preclinical studies. However, there is a need for more extensive studies to systematically evaluate how different molecular structures of NKCE impact immune synapse formation, pharmacokinetics, effector functions, and *in vivo* efficacy. Compared to T cell engagers, the current pool of NKCE formats is limited; hence, further exploration of additional NKCE formats is necessary to achieve the desired antitumor activities. Moreover, challenges such as potential on-target-off-tumor effects, NK cell exhaustion, and poor NK cell survival in the immunosuppressive TME have to be addressed during NKCE development (**Figure 3**). Therefore, further research is urgently needed to guide the development of NKCEs into off-the-shelf cancer treatment drugs.

## Author contributions

MZ, K-PL, and SX designed the work. MZ and SX wrote the manuscript. All authors contributed to the article and approved the submitted version.

## Glossary

**Table:**

ADCC	Antibody-dependent cellular cytotoxicity
ADCP	Antibody-dependent cellular phagocytosis
AICL	Activation-induced C-type lectin
AML	Acute myeloid leukemia
BCMA	B-cell maturation antigen
BiKE	Bispecific killer cell engager
CAR	Chimeric antigen-receptor
CDC	Complement-dependent cytotoxicity
CDR	Complementarity-determining regions
CHO	Chinese hamster ovary
CLEC12A	C-type lectin domain family 12 member A
CMV	Human cytomegalovirus
CRS	Cytokine release syndrome
CS1	CD2 subset-1
CTLA4	Cytotoxic T-lymphocyte–associated antigen-4
DAP10/DAP12	DNAX-activating protein of 10kDa/12kDa
DNAM-1	DNAX accessory molecule-1
EGFR	Epidermal growth factor receptor
EGFRvIII	Epidermal growth factor receptor variant III
Fab	Fragment antigen binding
Fas-L	Fas ligand
Fc	Fragment crystallizable region
FcγRIIIa	Fc γ receptor IIIa
FcϵRIγ	High-affinity IgE receptor γ
G-CSF	Granulocyte colony-stimulating factor
GPI	Glycosyl phosphatidyl inositol
Grb2	Growth factor receptor-bound protein-2
GVHD	Graft versus host disease
HEK	Human embryonic kidney
HER2	Human epidermal growth factor receptor-2
hGH	Human growth hormone
HLA	Human leukocyte antigens
IFN-γ	Interferon γ
IgG	Immunoglobulin G
IL	Interleukin
ILC	Innate lymphoid cell
IS	Immune synapse
ITAM	Immunoreceptor tyrosine-based activation motif
ITSM	Immunoreceptor tyrosine-based switch motif
ITT	Ig tail-tyrosine
kDa	Kilodalton
KIR	Killer immunoglobulin-like receptor
KIR2DS/KIR3DS	Killer cell immunoglobulin like receptor, two/three Ig domains and short cytoplasmic tail
mAb	Monoclonal antibodies
MHC I	Major histocompatibility complex class I
MICA	MHC I-related chain A
MM	Multiple myeloma
NCR	Natural cytotoxicity receptor
NK	Natural killer
NKCE	Natural killer cell engager
NKG2A	Natural killer group 2A
NKG2C	Natural killer group 2C
NKG2D	Natural killer group 2D
Nkp30	Natural cytotoxicity receptor-3
NKp46	Natural cytotoxicity receptor-1
Nkp80	Killer cell lectin-like receptor subfamily F member-1
NSG	NOD scid γ
OMCP	Orthopoxvirus major histocompatibility complex class I-like protein
PD-1	Programmed cell death protein-1
PD-L1	Programmed cell death protein-1 ligand
PEG	Polyethylene glycol
PI3K	Phosphatidyl-inositol-3-OH kinase
PKC	Protein kinase C
PLC	Phospholipase C
PVR	Poliovirus receptor
SAP	Signaling lymphocytic activation molecule–associated protein
scFv	Single-chain fragment variable
SEED	Strand-exchange engineered domain
SHP	SH2 domain-containing protein tyrosine phosphatase
SLAM	Signaling lymphocytic activation molecule
TAA	Tumor associated antigen
TACTILE	T cell activation, increased late expression
TIGIT	T cell immunoreceptor with Ig and ITIM domains
TIM3	T cell immunoglobulin and mucin domain-3
TME	Tumor microenvironment
TNF-α	Tumor necrosis factor alpha
TRAIL	TNF-related apoptosis-inducing ligand
TriKE	Trispecific killer cell engager
Vav	Vav guanine nucleotide exchange factors
VHH	Variable heavy domain of heavy chain
